# Gonadal Function and Reproductive Health Challenges in Triple a Syndrome: A Narrative Review

**DOI:** 10.3390/medicina61112073

**Published:** 2025-11-20

**Authors:** Ana Gheorghe-Milea, Carmen Emanuela Georgescu

**Affiliations:** 1 5th Department of Medical Sciences, Department of Endocrinology, Iuliu Hațieganu University of Medicine and Pharmacy, 400349 Cluj-Napoca, Romania; anamileagh@yahoo.com; 2 Endocrinology Department, Clinical County Emergency Hospital Cluj-Napoca, 400347 Cluj-Napoca, Romania

**Keywords:** triple a syndrome, allgrove syndrome, hypogonadism, sexual health, fertility, reproduction

## Abstract

*Background and Objectives:* Triple A syndrome (TAS) is a rare autosomal recessive disorder characterized by the triad of adrenocorticotropic hormone (ACTH)-resistant adrenal insufficiency, alacrimia (absence of tear production), and achalasia. This article aims to provide a better understanding of gonadal function and reproductive health in TAS by summarizing existing data from the literature regarding this topic. *Materials and Methods:* A comprehensive literature review was carried out until September 2025, using four electronic databases (PubMed, Embase, Web of Science and Scopus). *Results:* The review included a total of 25 articles. The most frequent findings in the articles included in the review were erectile dysfunction, ejaculatory dysfunction, delayed puberty, hypogonadism and fertility issues. Furthermore, several studies revealed reduced adrenal androgen levels in male patients, while other case reports documented the presence of osteoporosis in individuals with TAS. *Conclusions:* Gonadal dysfunction and reproductive health challenges in TAS remain underexplored aspects. The multisystemic nature of TAS requires a comprehensive approach to patient care for optimizing quality of life, and this review underscores the importance of evaluating reproductive function in individuals with this rare syndrome.

## 1. Introduction

Triple A syndrome (TAS) is a rare autosomal recessive disorder first described in 1978 [1], characterized by the classical triad of adrenocorticotropic hormone (ACTH)-resistant adrenal insufficiency [2], alacrimia (absence of tear production) and achalasia. This condition arises from mutations in the *AAAS* gene located on chromosome 12q13 [3], which encodes a 60 kDa WD-repeat-containing protein called ALADIN, whose precise function is currently unknown [4]. ALADIN is a component of the nuclear pore complex (NPC), a large multiprotein entity that mediates nucleocytoplasmic transport [5]. It is believed that the abnormal ALADIN proteins fail to target NPCs and are mainly localized into the cytoplasm, causing nuclear import defects, which in turn lead to increased susceptibility of cells to oxidative stress, cell death, and tissue degeneration [4,6,7].

TAS is characterized by a high degree of variability even in patients with the same mutation [8]. This lack of genotype/phenotype correlation highlights the complexity of the pathogenesis of TAS, with other factors probably playing an important role. While the primary characteristics of TAS are well-documented, patients frequently exhibit additional features, including manifestations caused by central, peripheral and/or autonomic nervous system impairment, optic atrophy, dysmorphic facial features, dental disorders, osteoporosis, short stature, microcephaly or palmar/ plantar hyperkeratosis [9,10,11,12,13,14]. Evidence from case reports and small studies suggests a range of reproductive health challenges in TAS patients (delayed puberty, menstrual irregularities, infertility or erectile dysfunction), with potentially profound implications for mental health, quality of life, and family planning.

This article aims to provide a better understanding of gonadal function and reproductive health in TAS by summarizing existing data from the literature regarding this topic.

## 2. Methods

A review of the literature was carried out until September 2025, using four electronic databases (PubMed, Embase, Web of Science and Scopus). Additionally, a manual search was conducted in Google Scholar and in the reference lists of the retrieved articles. We included human studies reporting data on gonadal development or function, the hypothalamic–pituitary–gonadal (HPG) axis, reproduction, sexual function, puberty, menstrual function, or fertility in patients with TAS. The following exclusion criteria were used: (1) unclear diagnosis of TAS, (2) evaluation of other endocrine pathologies, (3) studies on adrenal insufficiency alone without TAS, (4) book chapters, (5) articles in languages other than English, (6) studies performed on animals or cell cultures, (7) studies performed on a duplicate group of patients.

## 3. Results and Discussion

A total of 25 articles were included in this review. For each selected article, information on sample size, patient demographics, findings related to gonadal function or reproductive health, additional disease manifestations, and genetic analysis were extracted (Table 1). In several of the reviewed studies, data was unavailable for certain individuals; therefore, only patients with complete or partially available data were included in the table.

Gonadal dysfunction and reproductive health challenges in TAS remain underexplored aspects, although they carry significant implications for patients’ quality of life and long-term health outcomes. Several factors may account for the limited understanding of these entities in TAS: (1) the rarity of the condition, which results in its characterization being predominantly based on case reports and case series, restricting a comprehensive understanding of its full spectrum, (2) the fact that most reported cases involve children, with fewer studies on adolescents and adults, (3) the syndrome’s highly variable presentation, (4) sociocultural stigmas, personal discomfort, or the sensitive nature of these topics, which may discourage patients from discussing them openly and (5) the fact that the clinical focus is often directed toward the more severe, hallmark features, leaving secondary issues like gonadal dysfunction underreported and overlooked, highlighting a need for more targeted research.

### 3.1. Gonadal Function and Puberty

Delayed puberty (DP), a frequent finding in the reviewed cases, is defined in boys as the absence of testicular enlargement by the age of 14, or more than 5 years from onset to completion of genital development. In girls, it represents the absence of thelarche at the age of 13, over 5 years between thelarche and menarche, or no menstruation by the age of 16 [40,41].

A representative example is the patient presented by Cherif Ben Abdallah et al. [18] who was diagnosed with hypogonadotropic hypogonadism after being investigated for delayed puberty. At the age of 18 years, he exhibited G1P1A1 pubertal development, micropenis, gynecomastia and decreased levels of luteinizing hormone (LH) and total testosterone. The gonadotropin-releasing hormone (GnRH) test was performed and the LH and follicle-stimulating hormone (FSH) peaks after stimulation were 8 and 7 mU/mL, respectively, indicating a hypothalamic origin of hypogonadism. However, the magnetic resonance imaging (MRI) did not reveal any pathological aspects. Genetic analysis was performed on the *AAAS* gene and seven additional genes known to be associated with congenital hypogonadotropic hypogonadism. A homozygous mutation (c.1232+1G>C) was found in the *AAAS* gene, while the sequencing of the *PROP1*, *GnRHR*, *TAC3*, *TACR3*, *PROK2* and *PROKR2* genes failed to identify any mutations. A homozygous intronic variation (c.244+128C>T; dbSNP: rs350129) was found in the *KISS1R* gene, but in silico analysis did not indicate any evidence of a deleterious effect. The authors concluded that evaluation for hypogonadotropic hypogonadism is warranted in patients with TAS carrying the c.1232+1G>C mutation [18].

Considering the high expression of ALADIN in the pituitary gland [3], hypogonadism in TAS might stem from hypothalamic/pituitary dysfunction. Corroborating evidence for this hypothesis is provided by a separate case report describing a 15-year-old male with TAS assessed for delayed puberty [16]. The basal levels of LH and FSH were 0.1 and 3.3 IU/l, respectively, with a normal, pubertal response to GnRH administration. His puberty started spontaneously at the age of 17 years, but when he was reevaluated 4 years later, he displayed Tanner stage 3 development, his testosterone levels were low, and gonadotropins were at the lower end of the reference range. The authors concluded that the patient’s delayed and slowly progressing puberty could reflect a gradual impairment of the pituitary function and suggested that testosterone replacement therapy might be beneficial [16]. However, Bustanji et al. [38] documented a case of delayed puberty in a male patient who did not demonstrate clinical improvement following testosterone therapy. The patient was initially assessed at 16 years of age, presenting with absence of secondary sexual characteristics and prepubertal levels of FSH and LH. Despite receiving testosterone supplementation, no significant pubertal development was observed. The authors suggested that the delayed puberty might be related to the loss of function in the ALADIN protein. Notably, the patient had a cousin with TAS who did not experience pubertal delay [38].

Gonadal dysfunction in association with TAS was also mentioned in the case reported by Nakamura et al. [30]. Additionally, another study described a female patient with a complex neurological presentation and features consistent with gonadotropic dysfunction including delayed menarche, early menopause, and reduced fertility [39].

The systemic effects of the syndrome, including fatigue, malnutrition, and overall poor health might be involved in the pubertal delay by suppressing HPG axis, similar to other chronic illnesses, causing functional hypogonadotropic hypogonadism. Vezzoli et al. [37] reported the case of a patient who, at the age of 14 years, presented with significant weight loss and fatigue and was diagnosed with primary adrenal insufficiency (PAI). At that time, he exhibited Tanner stage 1 development. After starting glucocorticoid replacement therapy, he showed significant clinical improvement, and puberty began spontaneously one year later [37].

Normal pubertal development in patients with TAS was also reported by several authors [17,21,24,27,28,32,33,34]. Precocious puberty, defined by the first signs of puberty developing before 8 years in females and 9 years in males, was identified in two Romanian patients carrying the p.Ser263Pro mutation. This is one of the most common mutations found in the *AAAS* gene and appears to be more prevalent among Slavic populations [33].

### 3.2. Erectile and Ejaculatory Dysfunction

Penile erection is a spinal reflex that involves vascular, psychogenic, neurogenic and endocrine mechanisms, highlighting the complex nature of erectile dysfunction (ED), the most frequent finding in the reviewed articles [42].

Several mechanisms might account for the development of ED in TAS patients. Testosterone plays a critical role in libido and nocturnal erections [43] and ED is often associated with hypogonadism, as well as other endocrine disorders such as hyperprolactinemia or thyroid dysfunction [44]. Psychogenic ED, once thought to be the most prevalent cause of ED, is now understood to frequently co-occur with physical factors, such as vascular insufficiency and neurological conditions [45]. The psychological impact of TAS and its complications might be an important component of ED in these patients. Moreover, peripheral sensory neuropathy and autonomic neuropathy are common features described in association with TAS [12], which could contribute to the development of ED. Oxytocin, dopamine, serotonin and ACTH might play a role in the central regulation of the erectile process [42]. Experimental data revealed that administration of ACTH–melanocyte-stimulating hormone peptides in rats facilitated penile erection, ejaculation, and copulatory behavior [44,46]. Considering that ACTH insensitivity is regarded as a key mechanism for adrenal insufficiency in TAS [47], it can be hypothesized that this resistance has implications for the development of ED as well in these patients. Additionally, ED was described in association with Addison’s disease [48] and improved significantly after the initiation of replacement therapy, although it should be noted that the etiology of the PAI was autoimmune in the cases that were evaluated.

In one case report included in the present review, ED and absence of morning erections for six months were described in a patient who exhibited normal testicular volume, fully developed secondary sexual characteristics, normal libido, and normal levels of prolactin, LH and FSH [15]. The patient was also diagnosed with orthostatic hypotension (a condition associated with autonomic dysfunction) and peripheral sensory neuropathy. Other TAS cases included in this review also demonstrated the co-occurrence of erectile dysfunction and orthostatic hypotension [19,20,22,24,28,29,36,39]. The testosterone levels were determined in two of these patients [19,22] and found to be within normal range. In another case report [25], the patient had normal libido but complained of sexual impotence. The blood tests showed low levels of both total and free testosterone, which were attributed to the use of high-dose prednisone for the past 12 years, prescribed by neurologists for dysimmune neuropathy. The ED was considered to be either a result of the low testosterone levels or a potential indication of dysautonomia [25].

Ejaculatory dysfunction encompasses a range of conditions, from premature ejaculation to delayed ejaculation, anejaculation, as well as retrograde ejaculation. Ejaculatory dysfunction was another complaint reported in association with ED in patients with TAS [23,24,28,29,35]. Two of these reports emphasized the proper sexual development of the patients [23,24]. The majority of studies revealed anejaculation, only one patient exhibiting retrograde ejaculation [29].

The ejaculatory process is primarily controlled by the autonomic nervous system [49]; however psychological and hormonal factors (low levels of testosterone, high levels of prolactin) also play a significant role in the condition [50]. Similarly to ED, psychological distress resulting from the disease, along with hypogonadism and dysautonomia, could contribute to ejaculatory dysfunction in patients with TAS. All the male patients evaluated by Dumic et al. [28] exhibited erectile and ejaculatory dysfunction and neurological disorders. They had normal pubertal development and libido, normal levels of gonadotropins, testosterone and inhibin B, normal testicular morphology and low levels of adrenal androgens. The authors also emphasized that other possible causes for ED were excluded. Only one patient reported weak morning erections that diminished over time and some patients recalled occasional nocturnal and daytime erections during adolescence, which became rare or absent in adulthood. Development of complete ED in the oldest patients of the cohort was considered by the authors to be consistent with the progressive course of neurological impairment, particularly dysautonomia in TAS. Therapy with sildenafil was used in four patients, but response was limited (weak erections, without ejaculation) [28].

### 3.3. Fertility

In the study by Dumic et al. [28], assessment of fertility among six male patients was constrained by ejaculatory dysfunction and by their refusal to undergo testicular biopsy. However, the authors presumed that spermatogenesis was preserved and attributed the absence of offspring to sexual inactivity consequent to sexual dysfunction. The same study included a 44-year-old woman diagnosed with TAS who successfully conceived and delivered at the age of 29 years, following an uneventful pregnancy. A subsequent conception five years later resulted in miscarriage during the sixth week of gestation. Her anti-Müllerian hormone concentration was low, consistent with diminished ovarian reserve. In a separate report, Kimber et al. [39] documented reduced fertility, delayed menarche and early menopause in a female patient with TAS; similar manifestations were noted in the patient’s sister, whose TAS status was not reported and who died of ovarian carcinoma in her fifth decade. Azoospermia was documented by Agarwal et al. [20] in a 46-year-old male patient with TAS and erectile dysfunction.

Experimental data indicate a potential role for ALADIN in maintaining normal fertility. Huebner et al. evaluated mice lacking the *AAAS* gene, revealing that females were sterile, while males remained fertile [51]. Follicular development and ovulation occurred normally both in wild-type and ALADIN-deficient mice [51]. Taking into consideration the findings of a previous study [52] which showed that ALADIN depletion impairs mitotic spindle assembly, Carvalhal et al. [53] hypothesized that ALADIN might also play a role in meiosis, which could explain infertility in female mice lacking the *AAAS* gene. They showed that oocytes from these mice failed to extrude the polar body due to defects in meiotic spindle positioning and even those which successfully ejected the polar body had a reduced rate of fertilization (11.4%), while the obtained embryos were unable to progress past the two-cell stage [53]. Consequently, it could be speculated that some entities described above (ED, ejaculatory dysfunction or hypogonadotropic hypogonadism) might lead to infertility in TAS, but mutations in the *AAAS* gene could also have a direct impact on gametogenesis, fertilization or embryonic development.

### 3.4. Adrenal Androgens

Several male patients from the reviewed cases [16,19,28] with complaints regarding gonadal function or sexual health were found to have low levels of adrenal androgens. Resistance to ACTH could account for this deficiency in patients with TAS. In a study that included 23 patients with this syndrome [13], DHEA-S levels were low in all participants and the authors hypothesized that the increased vulnerability of the reticularis zone to oxidative damage was responsible for this finding. Adrenal androgen insufficiency determines absence of adrenarche and might contribute to other disorders such as reduced libido, sexual dysfunction (particularly in women), fertility issues, low bone mineral density, muscle weakness, and mood disorders [13]. However, two patients (one female and one male) with low androgen levels presented in this review were asymptomatic [26,27]. One study suggested that DHEA supplementation could be recommended to TAS patients for improvement of their general health and well-being [9].

### 3.5. Osteoporosis

Beyond its direct impact on reproductive health, gonadal dysfunction in TAS could also contribute to broader systemic complications, notably impaired bone health. The presence of osteoporosis in patients with TAS was reported in several studies included in this review [16,26,28], as well as other reports from the literature [54,55]. However, in the study by Bizzarri et al. bone mineral density (BMD) was normal [34]. Apart from hypogonadism, several other mechanisms might account for the reduction in BMD in these patients: low levels of adrenal androgens, reduced physical activity and sun exposure in patients with debilitating neurological manifestations, overtreatment with glucocorticoids or malnutrition resulting from achalasia [12]. It was suggested that this disorder is frequently overlooked in TAS and that screening should be performed systematically, along with education on preventive measures and appropriate nutritional supplementation options [12,55]. Dumic et al. even hypothesized that considering the ubiquitous expression of ALADIN, early development of osteoporosis might represent a primary manifestation of TAS [55].

### 3.6. Genotype-Phenotype Correlations

Pogliaghi et al. [56] examined the genetic and phenotypic diversity of TAS and concluded that pathogenic variants occur across all 16 exons, without evident mutational hotspots and thus, comprehensive diagnostic evaluation should involve sequencing the full gene, with particular attention to intron–exon boundaries. The authors also noted that current evidence fails to establish a clear genotype–phenotype relationship in TAS [56]. However, other authors observed differences when comparing phenotypes in TAS patients with truncating (nonsense, splice-site, or frameshift mutations) and non-truncating (missense) mutations. Ikeda et al. reported that individuals with at least one missense allele had milder disease and later onset of motor impairment than those with biallelic truncating variants [57]. According to Patt et al., patients harboring truncating mutations exhibited an increased prevalence of adrenal insufficiency, while those with non-truncating variants more commonly demonstrated neurological dysfunction, with no significant group differences for achalasia or alacrimia [58].

**Figure 1:**
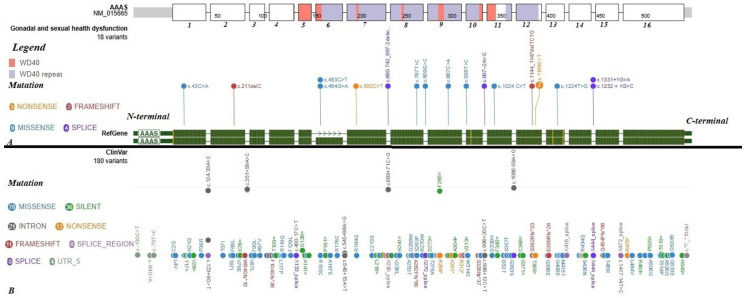
Lollipop plot of mutations in the *AAAS* gene (NM_015665; hg38). The upper panel (**A**) presents variants in the *AAAS* gene reported in patients with gonadal or sexual health dysfunction. The lower panel (**B**) includes a summary of *AAAS* variants reported in the ClinVar database [59]. The mutations documented in the reviewed cases are not evenly distributed: the majority localize to 3′ exons encoding the ALADIN WD40-repeat region, with additional canonical splice-donor mutations at adjacent intron–exon junctions in the same interval; comparatively few occur in 5′ exons (one missense and one frameshift mutation). No clear mutational hotspot was apparent. Lollipop plots were prepared based on the images generated with ProteinPaint [60].

We examined whether a distinctive positional pattern exists among *AAAS* variants associated with gonadal or reproductive health dysfunction. Figure 1 presents the pooled distribution derived from the literature synthesized in this review alongside the full spectrum of mutations included in the ClinVar database [59]. A regional enrichment of variants was apparent in the C-terminal portion of the *AAAS* gene, particularly within exons encoding the ALADIN WD40-repeat domain and at canonical splice sites in the same interval. This pattern may reflect a domain-specific susceptibility in ALADIN that could impair nuclear-pore–related functions relevant to the HPG axis and steroidogenesis. Although the number of cases is limited and subject to publication bias, with genetic data unavailable in some reports, the clustering argues against a uniform distribution of pathogenic changes and supports prioritizing C-terminal WD40-repeat and splice-region variants for testing and interpretation in future studies assessing gonadal and sexual function in TAS. Regarding genotype–phenotype correlations, erectile dysfunction was observed more frequently in patients harboring at least one missense variant in the *AAAS* gene, whereas delayed puberty was noted in individuals exhibiting truncating variants.

With respect to the other atypical manifestations noted in patients with TAS, the following genotype-phenotype patterns emerged from the reviewed cases. Osteoporosis was identified in two patients harboring nonsense variants [16,28], one of whom had severe skeletal involvement with multiple fractures [16]. This pattern could suggest that a higher loss-of-function burden may exacerbate bone fragility. Neuromuscular and ocular manifestations occurred frequently and exhibited substantial heterogeneity. Neurological dysfunction and orthostatic hypotension were frequently observed in individuals harboring at least one missense allele. Attention-deficit/hyperactivity disorder (ADHD) was documented in a patient homozygous for a canonical splice-donor variant (c.1331+1G>A) [27]. Skin involvement (e.g., palmar–plantar hyperkeratosis), myopathy, and ocular manifestations were more common among patients with non-truncating variants; however, these manifestations were also reported by Bustanji et al. [38] in a patient bearing a complex insertion–deletion (indel) [38]. Carriers of truncating variants were more likely to exhibit short stature and dysmorphic features, as well as renal and urinary tract manifestations. However, these associations are provisional given limited, heterogeneous data and require validation in larger cohorts.

### 3.7. Limitations

This review has several limitations. The available literature on gonadal function and sexual health in patients with TAS is scarce, consisting mainly of case reports, case series and small cross-sectional or retrospective studies with heterogeneous methodologies. Moreover, the evaluation of sexual health and gonadal function across reported cases involved diverse approaches, limiting comparability of findings. Confounding factors such as adrenal insufficiency, chronic glucocorticoid therapy, neurological dysfunction, and psychosocial influences further complicate attribution of findings specifically to the syndrome. In addition, the narrative nature of this review does not allow for systematic evaluation of study quality or risk of bias. Despite these challenges, this study continues to constitute a valuable contribution to the fields of endocrinology and genetics by drawing attention to the potential impact of TAS on the reproductive function and provides clinicians with a framework to recognize and address issues that may otherwise be overlooked in clinical practice. In performing so, this review contributes to improving awareness, clinical care, and long-term quality of life for affected patients.

## 4. Conclusions

This study underscores the importance of evaluating reproductive function in patients with TAS, a condition that appears to be associated with diverse impairments in sexual health, including erectile and ejaculatory dysfunction, delayed pubertal development, and infertility. From a genetic standpoint, we observed enrichment of variants associated with these manifestations in the WD40-domain exons and nearby canonical splice sites of the *AAAS* gene. Adrenal androgen insufficiency in TAS represents an additional concern, given its established association with sexual dysfunction and decreased libido, particularly in female patients. Moreover, osteoporosis may represent a significant manifestation of the syndrome, plausibly driven by both intrinsic disease mechanisms and secondary factors (e.g., hypogonadism). Overall, the multisystemic nature of TAS requires a comprehensive approach to patient care for optimizing quality of life in individuals with this rare syndrome.

## Figures and Tables

**Table 1:** Gonadal function and sexual health in Triple A syndrome patients.

First Author (Year) and Reference	T	Country	N	Age (y)	Sex	Findings Related to Gonadal Function and Sexual Health	Classical Features			Molecular Genetic Findings	Other Manifestations
							AC	AL	PAI		
Sanyal (2013) [15]	R	India	1	22	M	Erectile dysfunction, loss of spontaneous morning erections	+	+	+	NA	Orthostatic hypotension, bilaterally diminished ankle reflexes, peripheral sensory neuropathy
Dusek (2006) [16]	R	Croatia	1	21	M	Delayed puberty	+	+	+	Heterozygous: c.580C>T (p.R194X) nonsense mutation and c.1159C>T (p.Q387X) nonsense mutation	Dysmorphic facial features (elongated face, thin upper lip), anisocoric pupils, amblyopia of the right eye, carious teeth, nasal speech, orthostatic hypotension, essential tremor, peripheral sensory-motor neuropathy, severe osteoporosis- history of right femoral fracture and forearm fracture
Akbaş (2021) [17]	R	Turkey	1	14	M	At the age of 14 y: Tanner stage 5 development; hypogonadism excluded by laboratory evaluation	+	+	+	Homozygous: c.1368_1372delGCTCA	-
Cherif Ben Abdallah (2014) [18]	R	Tunisia	1	18	M	Delayed puberty, hypogonadotropic hypogonadism	+	+	+	Homozygous: c.1232+1G>C splice-donor mutation	Short stature
Nakamura (2010) [19]	SC	Japan	1	60	M	Sexual impotence	+	+	−	Homozygous: c.464G>A (p.R155H) missense mutation	Optic atrophy, nasal speech, furrowed tongue with fasciculation, orthostatic hypotension, distal motor deficit and amyotrophy, spastic gait, peripheral sensory neuropathy, hyperreflexia of deep tendon reflexes (except for ankle reflexes, which were abolished), pes cavus, bilateral extensor plantar responses
Agarwal (2021) [20]	LE	India	1	46	M	Erectile dysfunction, azoospermia	+	+	NA	Homozygous: c.43C>A (p. Q15K) missense mutation	Nasal speech, orthostatic hypotension, proximal muscle weakness, atrophy of the intrinsic hand muscles and tongue, hyperreflexia of deep tendon reflexes, bilateral extensor plantar responses
Moore (1990) [21]	CS	Canada	8	P VIII-3: 26	P VIII-3: M	P VIII-3: Normal sexual development	P VIII-3: +	P VIII-3: +	P VIII-3: +	NA	P VIII-3: Nasal speech, dysmorphic features, intellectual impairment (QI = 66), orthostatic hypotension, proteinuria, bilateral partial duplication of the renal collecting systems
de Freiatas (2018) [22]	R	Brazil	1	25	M	Erectile dysfunction	+	+	−	Heterozygous: c.938T>C missense mutation and c.1144_1147delTCTG frameshift mutation	Orthostatic hypotension, atrophy of the intrinsic hand muscles, atrophic tongue with fasciculations, abolished deep tendon reflexes in the lower limbs
Chu (1996) [23]	CS	USA	2	13	F	Sexually mature, well-proportionate	+	+	-	NA	Nasal speech, dry mouth, pallor of the optic disk, severe color blindness, slight ptosis, alternating diarrhea and constipation, decreased sweating, orthostatic hypotension, decreased heart rate variability, generalized hypotonia, unsteady gait, atrophy of the intrinsic hand muscles, bilateral pes cavus with thin, stork-like legs, hyperreflexia and bilateral extensor plantar response, peripheral sensory-motor neuropathy
				16	M	Inability to ejaculate	+	+	−	NA	Mild myopia and ptosis, pallor of the optic disk, severe color blindness, anosmia, nasal speech, dry mouth, periodontal disease, multiple caries, diminished facial expression, orthostatic hypotension and decreased heart rate variability, learning disabilities, unsteady gait, atrophy of the intrinsic hand muscles, bilateral pes cavus, hyperreflexia and bilateral extensor plantar responses, peripheral sensory-motor neuropathy
Pedreira (2024) [24]	R	Australia	1	37	M	Erectile dysfunction, ejaculatory failure	+	+	+	NA	Nasal speech, optic atrophy, orthostatic hypotension, mixed motor neuron abnormalities, predominantly distal muscle atrophy, bilateral pes cavus, hyperreflexia and extensor plantar responses
Gilio (2007) [25]	R	Italy	1	37	M	Sexual impotence	+	+	+	No mutations identified across *AAAS* exons	Optic atrophy, peripheral sensorimotor neuropathy, abolished deep tendon reflexes, marked distal muscle atrophy in the upper and lower limbs
Salmaggi (2008) [26]	R	Italy	1	33	M	Intact pituitary-gonadal axis	+	+	+	Homozygous: c.500C>T (p.A167V) missense mutation	Nasal speech, perioral fasciculations, thin upper lip, atrophy and fasciculations of the tongue, enamel hypoplasia, elongated face, bilateral optic disk atrophy, orthostatic hypotension, pectus carinatum, scoliosis, generalized muscular hypotrophy and weakness, diminished ankle reflexes, peripheral sensory-motor polyneuropathy, spastic gait, osteoporosis
Lu (2019) [27]	R	USA	1	14	F	Normal pubertal development, regular menstrual cycles	+	+	+	Homozygous: c.1331+1 G>A splice-donor mutation	Mild learning difficulty, ADHD
Dumic (2024) [28]	Cr-S	Croatia	6	Range: 19–48	M	Erectile and ejaculatory dysfunction, infertility (none had offsprings)	NA	P6: +	P6: +	P6: Heterozygous: c.887C>A (p. S263Y) missense mutation and c.1159C>T (p. Q387X) nonsense mutation	P6: Cutis anserina, palmoplantar hyperkeratosis, mouth dryness, glossitis, dental caries, hyperreflexia with diminished ankle reflexes, calves hypotrophy, pes cavus, unsteady gait, peripheral sensory-motor neuropathy, orthostatic hypotension, osteoporosis, permanent urinary catheter, chronic renal insufficiency requiring dialysis
			1	44	F	Normal pubertal development, regular menstrual cycles At the age of 29 y: Uneventful pregnancy At the age of 34 y: Miscarriage during the 6th week of gestation, diminished ovarian reserve	+	+	+	Homozygous: c.1024 C>T (p.R309X) nonsense mutation	Peripheral sensory-motor neuropathy, short stature
Vallet (2012) [29]	C	France	8	Range: 20–73	P1: F P2: M P3-8: 3M 3F	P1: Delayed puberty P2: Delayed puberty P3–P8: Impotence in one male patient, retrograde ejaculation in another male patient	All P: +	All P: +	All P: +	P1, P2: Homozygous c.1331+1G>A splice-donor mutation P3: Heterozygous c.352delT and c.1374–1176delTTCinsA mutations P4: Heterozygous c.991T>C (p.C331R) missense mutation and c.1331+1G>A splice-donor mutation P5: Heterozygous: c.1331+1G>A splice-donor mutation and c.1422G>C mutation P6: Homozygous: c.518A>T mutation P7: Heterozygous: c.43C>A (p. Q15K) missense mutation and c.928_931delGTCT mutation P8: Heterozygous: c.580C>T and c.1024C>T nonsense mutations	P1: Fissured, atrophic and paretic tongue, amyotrophy of all 4 limbs, cognitive symptoms, nasal speech P2: Fissured, atrophic and paretic tongue, pectus excavatum, pes cavus, Butler-Albright tubular acidosis, amyotrophy of all 4 limbs, cognitive symptoms, nasal speech P3–P8 (number of patients with the disorder): Walking difficulty (6), pyramidal syndrome and chronic peripheral neuropathy (6), telangiectasia and lower-limb edema (1), dyspnea (2), Achillean retraction (1) orthostatic hypotension (5), bladder dysfunction (3), diarrhea or constipation (3), dyshidrosis (2), cerebellar syndrome (2), cognitive symptoms (3), amyotrophy (5), nasal voice (velar insufficiency) (3), amyotrophic or paretic tongue (2), oropharyngeal dysphagia (5), facial motor deficit (3), ocular and ophthalmological signs (3), orthopedic deformation (3)
Nakamura (2018) [30]	R	Japan	1	31	M	Gonadal dysfunction	+	+	-	Homozygous: c.463C>T (p. R155C) missense mutation	Peripheral sensory-motor neuropathy
Razavi (2010) [31]	CS	Iran	4	Range: 6–15	P1, P2, P4: F P3: M	P1, P2: Delayed puberty	P1, P4: − P2, P3: +	All P: +	P1, P3, P4: + P2: −	NA	P1: learning difficulties, premature teeth loss, amyotrophy, short stature, facial dysmorphism (narrow upper lip and down–turned mouth), nasal speech P2: learning difficulties, poor school performance, hearing deficit, premature teeth loss, amyotrophy, dysmorphic facial features, nasal speech P3: facial dysmorphism (long thin face with long philtrum, narrow upper lip), mental impairment, dysarthria, orthostatic hypotension, short stature P4: learning difficulties, nasal speech, dysarthria, mental impairment, ataxia, dysmorphic features
Salehi (2005) [32]	R	USA	1	24	F	Menarche at 15 years, regular menstrual cycles	+	+	+	Heterozygous: IVSC14 +1 G> A splice-donor mutation and p. R155P missense mutation	Facial dysmorphism (elongated face and a narrow upper lip), orthostatic hypotension, unsteady gait, peripheral sensory-motor neuropathy, marked distal muscular atrophy, hyperreflexia of deep tendon reflexes, but abolished ankle reflexes
Milenković (2008) [33]	CS	Serbia	3	P1: 12 P2: 5 P3: 3	P1: F P2, P3: M	P1: Normal pubertal development, premenarchal, B3P2 Tanner stage	P1, P2: + P3: −	P1, P2, P3: +	P1: + P2, P3: −	Heterozygous: c.787T>C (p.S263P)) missense mutation and c.1261_1262insG mutation	P1: Poor coordination, limited dorsal flexion of the feet, muscle weakness, hyperreflexia, palmoplantar hyperkeratosis, abnormal dermatoglyphs P2: Hyperreflexia, convergent squint
	C	Poland, Czech Republic, Hungary, UK, Romania, Germany, Slovenia, Croatia, Serbia	17 families	NA	NA	2 P from Fam 5: precocious puberty 1 P from Fam 1: delayed puberty 1 P from Fam 14 and 1 from Fam 16: normal puberty	Fam 1, 5, 14 and 16: +	Fam 1, 5 and 16: + Fam 14: −	Fam 1, 5 and 16: + Fam 14: −	Homozygous: c.787T>C (p. S263P) missense mutation	Fam 1 (M): cutis anserina, poor wound healing, muscle weakness, hyperreflexia, ataxia, anisocoria, nasal speech, visual problems Fam 5: P1 (M): palmoplantar hyperkeratosis, poor wound healing, muscle weakness, hyperreflexia, ataxia, pes cavus, postural hypotension P2 (M): palmoplantar hyperkeratosis Fam 14 (F): pes cavus, nasal speech, visual problems Fam 16 (M): palmoplantar hyperkeratosis, cutis anserina, poor wound healing, muscle weakness, hyperreflexia, ataxia, anisocoria, nasal speech
Bizzarri (2013) [34]	R	Italy	1	13	M	Bilateral cryptorchidism, inguinal hernia, hypospadias At the age of 13: Tanner stage 2, bilateral TV of 6 mL	+	+	+	Homozygous: c.1224T>G (p.L381R) missense mutation	Mild mental impairment, dysarthria, nasal speech, malar hypoplasia and prognathism, horizontal gaze, nystagmus, bilateral mydriasis, mild bilateral optic atrophy, hyperkeratosis of elbows and knuckles, hypothenar prominence wasting, unsteady walking on toes, hyperreflexia of deep tendon reflexes, bilateral extensor plantar response, peripheral sensory-motor neuropathy, syringomyelia, type 1 Chiari malformation
Macke (2022) [35]	R	USA	1	29	M	Erectile dysfunction, inability to ejaculate	+	+	−	Heterozygous: c.211delC frameshift mutation and c.809G>C (p.R270P) missense mutation	Optic atrophy, distal amyotrophy, neuropathy
Bentes (2001) [36]	R	Portugal	1	36	M	Sexual impotence	+	+	+	NA	Orthostatic hypotension, dysphonia, dysarthria, left palatal paresis, decreased gag reflex, atrophic and spastic tongue, spastic tetraparesis, amyotrophy in all four limbs, brisk masseter and tendon reflexes, depressed ankle reflexes, increased truncal perspiration
Vezzoli (2020) [37]	CS	Italy	3	P1: 20 P2: 15 P3: 6	P1, P3: F P2: M	P2: Delayed puberty	All P: +	All P: +	All P: +	P1: Heterozygous c.43C>A missense mutation and c.1331+1G>A splice-donor mutation P2: Homozygous c.997–2A>G splice-acceptor mutation P3: Heterozygous c.43C>A missense mutation and c.765dupT frameshift mutation	P1: hyperreflexia, muscle weakness, nasal speech, ataxia, polyneuropathy, orthostatic hypotension, neurogenic bladder P2: hyperreflexia, muscle weakness, nasal speech, ataxia, polyneuropathy, P3: hyperreflexia, muscle weakness, nasal speech, ataxia
Bustanji (2015) [38]	R	Jordan	1	17	M	Delayed puberty	+	+	+	Homozygous: c.690-742_997-2delinsTGAGGCCTGCT mutation	Fissured and dry tongue, palmar and plantar hyperkeratosis, distal muscle atrophy, hyperreflexia, bilateral partial optic atrophy
Kimber (2003) [39]	CS	UK	3	P1: 40 P2: 45 P3: 60	P1, P3:M P2: F	P1: Erectile dysfunction P2: Gonadotropic dysfunction, delayed menarche, early menopause, reduced fertility P3: Erectile dysfunction	All P: +	P1, P2: + P3: NA	All P: −	NA	P1: Dysmorphic facial features, nasal speech, pupil mydriasis with an absent direct light response and poor response to accommodation, bilateral optic atrophy, bilateral palatal paresis with an absent gag reflex, small and spastic tongue, regional hyperhidrosis, orthostatic hypotension, ataxia, dysarthria, amyotrophy of all 4 limbs, bilateral extensor plantar responses, pes cavus, hyperreflexia of deep tendon reflexes with abolished ankle reflexes P2: Nasal speech, reduced direct and consensual pupillary light response, bilateral pallor of the optic disk, wasting and fasciculation of the tongue with palatal paresis, hyperreflexia of deep tendon reflexes, bilateral extensor plantar responses, bilateral pes cavus, ataxia, orthostatic hypotension P3: Right sided partial ptosis and miosis, scoliosis, ataxia, peripheral sensory neuropathy, hyperreflexia of deep tendon reflexes, bilateral extensor plantar responses, regional hyperhidrosis

T, article type; N, number of patients; y, years; AC, achalasia; AL, alacrimia; PAI, primary adrenal insufficiency; +, present; −, absent; NA, information not available; M, male; F, female; R, case report; CS, case series; LE, letter to the editor; SC, short communication; Cr-S, cross-sectional; C, cohort; ADHD, attention deficit hyperactivity disorder; P, patient; Fam, family; TV, testicular volume.

## Data Availability

Not applicable.
